# Validation of the Magnetic Stirrer Method for the Detection of *Trichinella* Larvae in Muscle Samples Based on Proficiency Tests Results

**DOI:** 10.3390/foods11040525

**Published:** 2022-02-11

**Authors:** Mirosław Różycki, Weronika Korpysa-Dzirba, Aneta Bełcik, Ewa Bilska-Zając, Maciej Kochanowski, Jacek Karamon, Jacek Sroka, Tomasz Cencek

**Affiliations:** Department of Parasitology and Invasive Diseases, National Veterinary Research Institute in Pulawy, Al. Partyzantow 57, 24-100 Pulawy, Poland; mrozycki@piwet.pulawy.pl (M.R.); aneta.belcik@piwet.pulawy.pl (A.B.); ewa.bilska@piwet.pulawy.pl (E.B.-Z.); maciej.kochanowski@piwet.pulawy.pl (M.K.); j.karamon@piwet.pulawy.pl (J.K.); jacek.sroka@piwet.pulawy.pl (J.S.); tcencek@piwet.pulawy.pl (T.C.)

**Keywords:** *Trichinella* spp., validation, magnetic stirrer method, proficiency test

## Abstract

*Trichinellosis* is a zoonotic disease caused by the nematodes of the genus *Trichinella*. Infection takes place through the consumption of infected meat containing live larvae. The only way to prevent the disease is to break its epizootic chain. To ensure effective control of *Trichinella* spp., a range of preventive and control measures have been undertaken. These efforts have been focused on controlling *Trichinella* in domestic pigs, the main source of the disease. Artificial digestion is also the reference point for other methods for *Trichinella* risk control. Descriptive data validation of the digestion assay was presented in 1998 based on results published by scientific laboratories. Herein, we supplement those data by characterizing the method’s performance in inter-laboratory comparisons. The source of data was the results of Proficiency Testing conducted in 2015–2019. Samples were contaminated by 0, 1, 3, and 5 larvae. In total, 7580 samples were examined by the laboratories. Based on Proficiency Testing results, the main parameters characterizing the method performance in field conditions were established as follows: specificity, 97.3%; sensitivity, 86.5%; accuracy, 89.2%; uncertainty, 0.3; limit of detection (LOD), 1 larva; and limit of quantification (LOQ), 3 larvae.

## 1. Introduction

**Figure 1:**
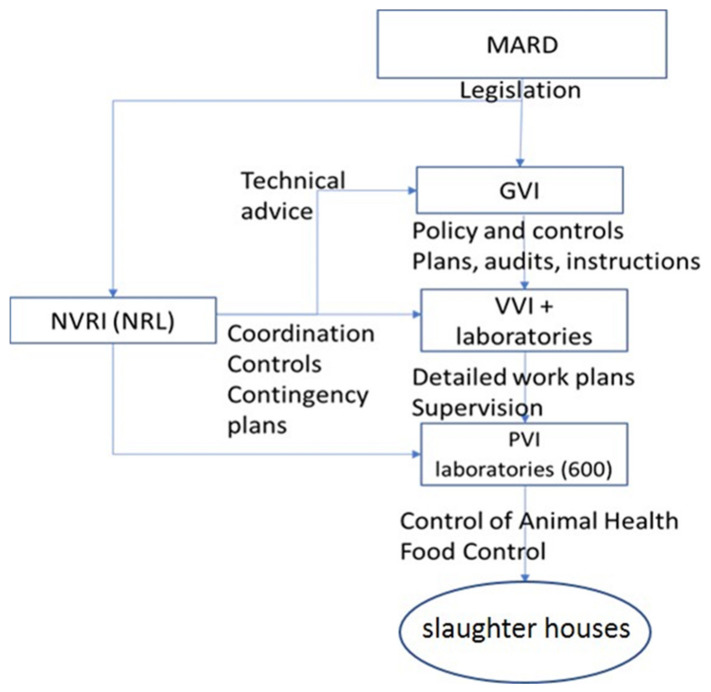
Control system for *Trichinella*. Ministry of Agriculture and Rural Development—MARD, General Veterinary Inspectorate—GVI, Voivode Veterinary Inspectorate VVI + laboratories (Regional Veterinary Laboratories ZHW), Poviat Veterinary Inspectorate—PVI + official laboratories, National Veterinary Research Institute (National Reference Laboratory)—NRVI (NRL).

The *Trichinella* genus (Phylum: Nematoda, Class: Enoplea, Order: *Trichocephalida*, Family: *Trichinellidae*) includes 13 species so far and additional genotypes that have not been recognized as distinct species [1,2]. Four species, namely, *Trichinella spiralis*, *Trichinella britovi*, *Trichinella nativa*, and *Trichinella pseudospiralis*, are known to be circulating in Poland in a wide spectrum of hosts [3,4,5,6]. The transmission of the parasite occurs when a susceptible host ingests muscle tissue containing live larvae [7]. Species such as foxes, raccoon, dogs, and wild boars are the natural reservoirs of the parasite [8,9], raising understandable concerns of the Veterinary Inspection (VI) and the consumer [10]. Examination of meat with the magnetic stirrer digestion (MSD) method is the most common and efficient form of preventing human *Trichinellosis* [11]. Using the MSD method, *Trichinella* larvae are detected from muscle tissue with an artificial digestion fluid and recovered under light microscopy in sediments according to Commission Implementing Regulation (EU) 2015/1375 of 10 August 2015, which lays down specific rules on official controls for *Trichinella* in meat. Currently, in Poland, over 3000 veterinarians and technicians in 600 laboratories provide examination for *Trichinella* spp. using the MSD method. The organization of the VI and the role of the National Reference Laboratory (NRL) for *Trichinellosis* is presented on the diagram below (Figure 1).

The VI is operationally divided into the General Veterinary Inspectorate (GVI), 16 Voivodship (Regional) Veterinary Inspectorates (VVI), and 305 Poviat (District) Veterinary Inspectorates (PVI). These are headed by Chief Veterinary Officer (CVO), Regional Veterinary Inspectors (VVI), and Poviat Veterinary Inspectors (PVI), respectively. The activity of the National Reference Laboratory for *Trichinellosis* (NRL) is focused on working with laboratories, organizing Proficiency Tests (PTs), and diagnostic and epidemiology of *Trichinella* spp. in animals [12,13]. The task of the VI is to ensure food safety [14]. A large group of technicians and veterinarians perform this task every day, and thanks to their work, there has not been a single outbreak of *Trichinellosis* in the past 10 years due to the consumption of meat tested by MSD. However, *Trichinella* spp. infections still occur as a result of consumption of untested meat, both pigs and wild boars, and Poland is one of the leading places in Europe for human *Trichinellosis* [15]. The scale of the problem is illustrated by 1030 cases (since the beginning of this century) with an average annual number of 51 and a median of 30. During that time, Poland was considered the fourth producer of pigs in Europe, with 908 slaughterhouses and 94 game-processing plants. Traditionally, veterinary laboratories for *Trichinella* in Poland are located by the slaughterhouses. Since 2009, all laboratories have been used for examination using the magnetic stirrer digestion method (MSD) [16]. In 2021, 362 laboratories were accredited in accordance with the International Standard Organization (ISO) Standard. The other laboratories, despite not having an accreditation certificate, also operate in accordance with the Quality Management System (QMS) [17,18].

According to the ISO definition, the term “quality” can be defined as, “The totality of features and characteristics of a product or service that bear on its ability to satisfy stated or implied needs” [19]. The applied tests should be characterized by high precision, accuracy, and repeatability of the results [20]. Therefore, the obligation of each laboratory is to take appropriate actions to confirm both the laboratory staff and the recipient of the laboratory test results in the belief that the method meets the quality requirements and is suitable for a specific application [13,21]. Primary validation for MSD was completed by Forbes and Gajadhar in describing the method in terms of official use needs [22]. In our study, we supplement and support those data by validation based on PT results. We collected data from 2015 to 2019 as results of PTs. For the PTs, we used both kinds of evaluation, qualitative and quantitative [17]. The organization of PTs for such a large scale requires the use of a method that will guarantee quick and easy preparation of samples while ensuring accuracy and repeatability. This was achieved with the use of a sample spiked with gelatin-coated larvae. The larvae released from the muscle tissue by the MSD method were harvested, rinsed in PBS, and embedded in 8% gelatin Type A (from pig skins in acid process, strength of 280–300 Bloom) used to protect the larvae. Each larva was protected with a 5 mm wall of gel, protecting larvae from drying and oxygen activity. Samples were delivered to laboratories in controlled conditions within 24 h post-preparation. Laboratories had to send their results online, and the evaluation was completed automatically. The partial PT reports were available for the laboratories within minutes after approving the sample results and sending the completed PT form. Each report contains information on the participant’s test results, evaluation of the results, and comparison with the previous test round. Results of examination of samples spiked with one larva were used to assess the performance within the entire region. Collected results of PTs enable us to use them as so-called field validation and establish parameters characterizing the method performed in the conditions of field laboratories.

## 2. Materials and Methods

### 2.1. Prerequisites

All laboratories performing meat inspection for *Trichinella* spp. were designated by an official veterinarian based on results of the audit from the Regional Veterinary Laboratory and training at the NRL. The capacity of the laboratory was confirmed according to the checklist provided by the NRL. Prior to designation, the performance of laboratories was confirmed by examination of diagnostic samples prepared by the NRL. The laboratories are obliged to document the course of examination in accordance with the principles of the quality system, taking into account critical points. Documentation forms, as well as Standard Operation Procedures (SOPs), are provided by VVL. Technical and metrological supervision over the equipment is carried out by the PVI. Chemical reagents for testing are controlled by the NRL; a list of approved suppliers is available on the GVI website [23].

### 2.2. Sample Preparation

Prior to PT, round test samples were prepared. Firstly, live larvae were obtained from the muscles of naturally infected wild boars. Each larva was embedded in 5 mm of gel, which protected the larvae from drying and oxygen activity. The larvae prepared this way are easy to count and add to a portion of minced meat. Each laboratory was provided with a set of samples. Each set contained four samples: negative, and three spiked samples (one larva, three and five larvae, respectively). Samples and laboratories were marked with unique codes. Results of PT were evaluated and published as regional reports. The methodology of sample preparation and evaluation criteria were published earlier [17]. Since the risk associated with horse meat consumption differs [23,24], laboratories examining horse meat and PT results in this type of meat were excluded from the study.

### 2.3. Data for Analysis

The data for analysis were PT results collected from 2015–2019 (Table 1). Within those five years, 1895 sets of samples were prepared and sent to the laboratories. The overall number of examined samples was 7580 and took into account the population proportion: 50% (standard), and the size of population, 7580, at least 5209 or more measurements were needed to have a confidence level of 99% so that the real value is within ±1% of the measured value. Outliers were excluded from the study (result that exceed 3 SD of the mean value). The number of participants taking part in PT research is variable; the highest number of laboratories participated in 2015 and the lowest in 2018. This can be explained by the fact that some laboratories are located at small family meat-processing plants (working periodically).

### 2.4. Matrix Variation

Matrix variation might be omitted since only minced pig meat (made from pork ham) was used for sample preparation [25]. To ensure high accuracy of the PT samples, the meat portions (50 g) were spiked with larvae embedded in gelatin. Small amounts of gelatin do not disturb the process of digestion. The clarity of the high bloom gelatin makes it easy to control the number of larvae added. The number of added larvae was checked twice before packing under the microscope (40× magnification, Olympus SZ40, Tokyo, Japan).

### 2.5. Sample Stability

Time, temperature, and sunlight are the main variables affecting the stability of laboratory samples. Their stability was confirmed using samples spiked with 5 larvae, and stability in gels was evaluated within 21 days of storage in the fridge at temperature 4–8 °C and examined every 3rd day. In addition, every round of PT was supported with extra sets of samples that were prepared and stored at 4 °C for 2 weeks to confirm the stability of samples used for PT study.

### 2.6. Sample Delivery, Time of Analysis, and Reporting

Samples were delivered to Voivode Veterinary Laboratory (VVL) under controlled conditions and were distributed to the field laboratories within 24 h. Each batch of PT samples was stored/transported under controlled conditions at 2–6 °C. Each shipment was accompanied by a thermoregistrator (LB-516T, LAB EL, Reguły Poland) recording start, end of mission, and the temperature with 1 min intervals, which allows us to trace back the conditions of transport. Until the analysis, field laboratories were required to store samples under controlled temperature of 2 to 8 °C. Laboratories were required to examine the set of PT samples within 5 working days. The results were sent online through forms available on PTs website [26]. After 10 working days, the time window for reporting results was closed. The form also includes additional questions for participants. These questions concerned the laboratory equipment, the type of glass used, the reagents, the number of dead larvae found during the examination, and the meat residues left on the mesh after digestion. This information was used to take appropriate protective and corrective actions in the situation of a non-compliant result obtained by the laboratory.

### 2.7. Sample Examination

Since joining the EU for official purposes, laboratories have only used the reference method described in Annex I in Commission Implementing Regulation (EU) 2015/1375 and the previous regulations [27]. The list of approved NRL reagents for MSD is available on the GVI website [28]. Delivered samples did not require any additional actions or processing. A detailed description of the procedure of PT sample examination was available for participants on the website.

### 2.8. Sample Evaluation

The results presented by the participants were assessed as qualitative and quantitative. In the qualitative examination, the results were assessed as being in conformity if *Trichinella* spp. larvae were found in positive samples but were not detected in samples without larvae. Results were defined as unacceptable when laboratories failed to detect *Trichinella* spp. larvae in samples that contained *Trichinella* spp. larvae, or they detected larvae in negative samples. Quantitative evaluation was based on guidelines given by the European Union Reference Laboratory (EURL). Absolute difference |Δ| between the reported results and the reference value was used for the quantitative assessment [12]. Depending on the value of the indicator |Δ| in quantitative evaluation, results are considered if:

|Δ| ≤ 2—‘satisfactory’.

|Δ| = 3—‘doubtful’.

|Δ| > 3—‘unsatisfactory’.

### 2.9. Reported Results

Within 2015–2019, 1895 laboratories participated in the organized PTs. The qualitative results of the sample examination are presented in Table 1.

During 2015–2019, the laboratories tested 7580 PT samples. In the qualitative study, the best percentage of correctly evaluated samples was achieved in 2017 (90%) and the worst was in 2018 (88%).

Differences in qualitative evaluation of the samples at different contamination levels are presented in the table below (Table 2).

According to the presented data, the highest number of incorrect results was recorded in the case of samples contaminated with one larva. However, as mentioned before, this level is the detection level of the method and is not generally used for laboratory evaluation. The distribution of PT results is presented in the form of graphs, where green color is used for correct, yellow for doubtful, and red for incorrect results.

**Figure 2:**
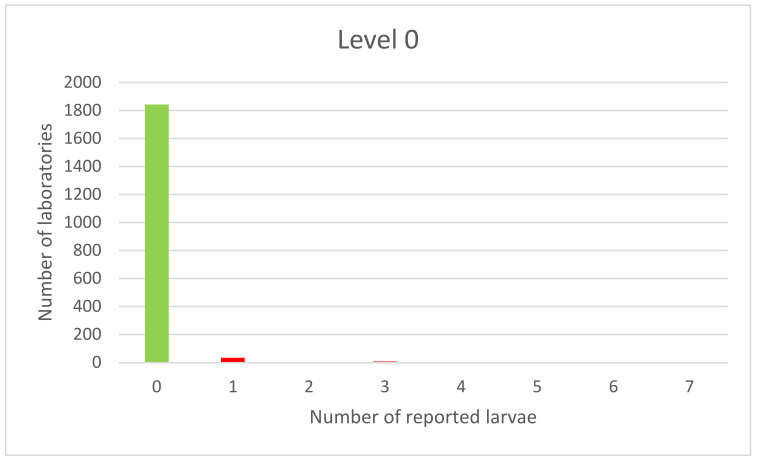
PT results for non-contaminated samples.

The results of the examination of *Trichinella* spp. free samples are shown in Figure 2.

**Figure 3:**
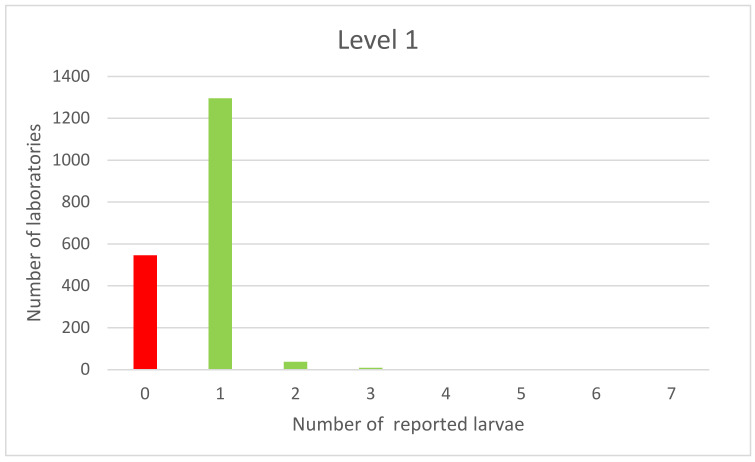
PT results of samples spiked with 1 larva.

The majority of laboratories reported consistent results. The distribution of the results is one-sided, normal for results of blank samples’ examination; however, several laboratories found at least one larva. The one-side distribution was also observed in the results of an examination of samples at the detection level (Figure 3).

The distribution of the test results for samples contaminated at the detection level is unimodal (one peak) with a left shift. At the detection level, *Trichinella* spp. larvae were not found by 547 laboratories; the results in compliance with the reference value were reported by 1296 laboratories. Three laboratories reported the detection of five larvae.

**Figure 4:**
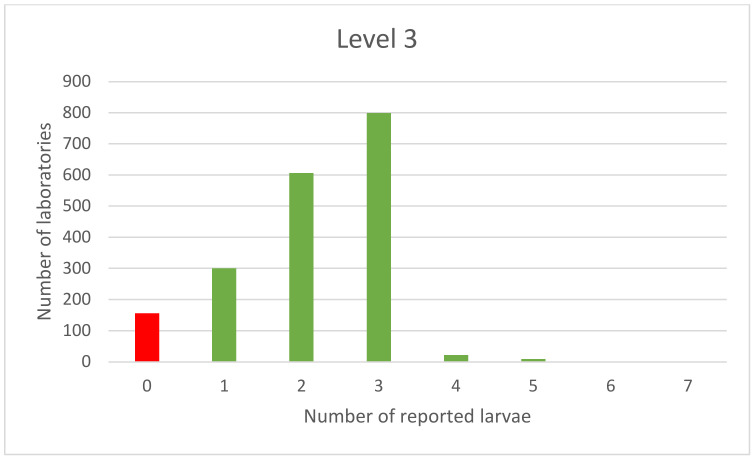
PT results of samples spiked with 3 larvae.

A more normal distribution can be observed for the results obtained with the samples contaminated with the three larvae, as presented in Figure 4.

The distribution of the results of the samples contaminated with three larvae indicates that only a few laboratories overshot the results by one larva. Compliance results were reported by almost 800 laboratories out of 1895 participating in the study.

**Figure 5:**
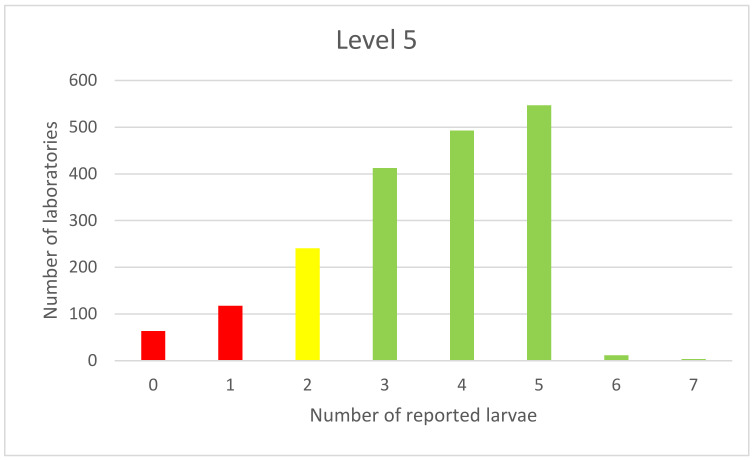
Distribution of PT results of samples spiked with 3 larvae.

The same pattern can be observed in the results of an examination of samples contaminated with five larvae (Figure 5).

The presented graph of the examination of samples contaminated with five larvae represents a one-sided type due to the average value being within the limit range.

PT results at all levels were statistically described in the form of a table. The statistical description is shown in Table 3.

As the table shows, kurtosis is positive for all tested levels; except level five, the intensity of the extreme values is greater than for the normal distribution.

Results of the quantitative evaluation are presented in Table 4.

The highest number of incorrect results were observed in samples spiked with one larva (detection limit), the lowest at level = 0.

The results of PTs described above were used to calculate the parameters characterizing the MSD in field laboratories. The table below shows the features that were specified for the MSD (Table 5).

## 3. Results

### 3.1. Precision

Precision is defined as compliance with the results of a series of measurements in a given sample using a given method. The measure of precision is the standard deviation (S) or the coefficient of variation, presented as a percentage for results over a series of measurements.

CV measures are often used as quality controls expressing the diversity of distribution (precision and repeatability). The coefficient of variation is a relative measure of dispersion, depends on the arithmetic mean, and is defined by the formula: where s denotes the standard deviation, x_mean_ ≠ 0. However, if the reference value is known, variation might be measured by simply averaging the standard deviation across the expected value, µ ≠ 0; both measures are given in Table 6.

The low values of CV inform that precision is high. The highest precision was obtained for samples spiked with five larvae (CV = 0.4), while the lowest was obtained with only one (CV = 0.73).

Reproducibility is defined as precision under reproducibility conditions, in different laboratories, with different technicians, over a long period, examined with the same method. Reproducibility was counted as the number of compliant results (reproduced) divided by the total number of results and then multiplied by 100%. To assess the reproducibility, results of the quantitative assessment were used.

In the case of using quantitative evaluation of reported results, the reproducibility will be slightly lower than qualitative evaluation but closer to the real performance of laboratories.

The limit of detection (LOD) for the MSD was established empirically as one larva per sample and was confirmed using the formula LOD = 3 s (based on large series of blind samples). The obtained result confirmed the empirical limit as metrologically correct (LOD = 3 s, LOD = 3 × 0.36 = 1.08).

The limit of quantification (LOQ) is understood as the lowest concentration of larvae that can be determined with an MSD with the specified accuracy and precision. It is a multiple of the detection limit, usually: LOQ = 3 LOD. Thus, the LOQ was established as 3.08 larvae per sample.

The specificity, sensitivity, and accuracy of the MSD performance by the laboratory were evaluated according to ISO 16140 Standard. The form sheet (Excel) that allows calculating the specificity (3.3), sensitivity (3.2), and accuracy (3.4) with a confidence interval for *p* = 95% was developed, with true positive results described as (PA), the number of false negatives described as (ND), true negatives described as (NA), and false positives described as (PD).

The confidence interval (CI) for *p* = 95% was also evaluated according to ISO 16140 Standard. The confidence interval (CI) for *p* = 95% was also evaluated according to the same document.

### 3.2. Sensitivity (SE)

The number of true positive results is (PA), that of false negatives is (ND).

Sensitivity confidence interval (86.0%, 97.6%).

(1) $$\mathrm{SE}=\frac{\mathrm{PA}}{\;\mathrm{PA}+\;\mathrm{ND}}\times 100\%=\frac{4915}{4915+770}\times 100\%=86.5\%$$

### 3.3. Specificity (SP)

The number of true negatives is (NA), and that of false positives is (PD).

Specificity confidence interval (96.9%, 97.6%).

(2) $$\mathrm{SP}\;=\frac{\mathrm{NA}}{\mathrm{NA}+\;\mathrm{PD}}\times 100\%=\frac{1843}{1843+52}\times 100\%=97.3\%.\;$$

### 3.4. Accuracy (AC)

The number of true positive results is (PA), and that of false positives is (PD).

Accuracy confidence interval (88.8%, 89.5%).

(3) $$\mathrm{AC}=\frac{\;\mathrm{PA}}{\mathrm{PA}+\mathrm{PD}}\times 100\%=\frac{4915}{4915+52}\times 100\%=89.2\%$$

### 3.5. Uncertainty 

Uncertainty was calculated according to the Guide to the Expression of Uncertainty in Measurement of ISO, which determines procedures in the uncertainty of the measurement. Since PT results may be considered a set of n-many independent measurements x1, x2, …, x*n* in an n-element sample, the data obtained during the examination of PT samples on level 5 were used. Uncertainty was calculated in a prepared Excel sheet using the formula: Uncertainty (u) = √ [∑ (xi − μ)2/(*n* × (*n* − 1))], where (xi) is the ith PT result, the mean value of PT results is (μ), and the number of results is (*n*) = 1895. The calculated uncertainty value for the samples at level 5 was 0.03.

## 4. Discussion

Food safety is defined as the set of conditions that must be met through activities that must be undertaken at all stages of food production to ensure food safety [29]. The production and marketing of safe food that does not harm the health and life of the consumer are one of the essential elements of the European Union’s policy [30]. Equality of laboratory methods is one of the priority measures within the European Community [31]. To achieve this goal, it is necessary to improve the training system for employees of the official food control. An important element of the system was the building of institutions of the NRL to enforce solutions consistent with the Community [12,24]. Ensuring harmonization of laboratory methods on the national level within the European Union and its effective implementation in practice is the main task of the NRL [12,32]. Since January 2006, Commission Regulation (EC) No. 2075/2005 of 5 December 2005 was introduced into Polish legislation [29]. This regulation indicated pooled sample digestion with the assistance of a magnetic stirrer, as a reference method and as a reliable method for routine use. The purpose of this regulation was to implement the digestion method in the same manner as it is in all EU countries. This task was given to the European Reference Laboratory for Parasites (EURLP) that was designated to coordinate the work of National Reference Laboratories (NRL) and uniform implementation of the method before the end of 2009. The National Veterinary Research Institute in Pulawy was designated by MARD in 2004 to ensure harmonization of MSD and speed up its implementation in practice. The MSD method was introduced in Poland in the same manner in all laboratories performing MSD, enabling to use the results obtained by field laboratories as the source of information for description of validation parameters based on PT results. Since 2005, the NRL in Pulawy has organized the PT on MSD; however, at that time, there was no established PTs methodology within the EU. The main problem was related to ensuring the stability of the samples during transport while maintaining an accurate regime of counting larvae [22]. That goal was impossible to achieve using naturally infected material due to the random localization of larvae in muscle tissue [32]. Even if material for PT samples was homogenized, reported results varied. This significantly hampered the assessment of the laboratories’ performance. The development of the methodology of sample preparation using partial digestion of muscle tissue allowed for more accurate analyses [32]. In 2012, we elaborated a method of larvae preservation with gelatin cover. Coating isolates larvae from the toxic oxygen effect and provides the proper moisture level, protecting larvae from drying. Since 2015, the one scheme for PT was used, and collected results provide field validation data [26]. The validation problem is generally underestimated, although many laboratories have completed a kind of validation in laboratory conditions [21,33]. The importance of foodborne parasitoses is widely recognized and often regulated by food safety standards [34,35]. Among many other harmful and dangerous parasites for humans, only in the case of *Trichinella* spp. each animal intended for consumption is subject to laboratory tests [35,36]. Increases in the number of human *Trichinellosis* outbreaks, despite increased testing requirements, have raised concerns about the validity of currently used assays and the reliability of test results, emphasizing the need for proper quality assurance (QA) in the test systems [13]. Currently, parasitological methods are analytically reviewed for validation by the Technical Subcommittee ISO/TC 34/SC 9/WG 6—Foodborne parasites. Even an established method such as an MSD lacks complete information. Generally, there is a problem with guidelines for validation of parasitological methods, and those established for bacteria are generally unsuitable for parasites [37]. Even the procedure used in ISO 16140 Standard was established for bacteria. Validation and standardization of methods help to understand the foodborne route of transmission, improve risk assessments, and help to identify and verify critical control points [10]. The MSD method is the first one described in ISO Standards [28] Thus far, validation processes have been carried out in laboratories selected for evaluation, which could slightly distort the image of the overall performed tests. Moreover, these laboratories knew that they were taking part in a scientific experiment, often for the first time, which could have resulted in additional staff involvement and performing tests with special care. A similar risk is also associated with Proficiency Tests; however, systematic participation in PTs reduces stress, and staff involvement is much lower. Therefore, we believe that compared to the tests conducted formerly, our research is more similar to the working conditions of field laboratories. Validation is also required in terms of the equality of methods. According to the recommendations of the International Commission on *Trichinellosis* (ICT), new alternative test methods must be at least as sensitive as MSD. Admission of new methods requires comparative tests. Previously described validation data from swine indicated that the currently accepted sample size of 1 g from individual carcasses consistently detected larval loads of at least 3 larvae per gram [38]. Larval loads of 1.0 to 1.9 larvae per gram required 3 to 5 g samples of muscle tissue for reliable detection [33]. Our study describes basic parameters characterizing the MSD, such as specificity, sensitivity, accuracy, repeatability, and uncertainty. Obtained results might be useful in the recognition of laboratories that meet the internationally accepted guidelines, especially now, when accreditation by an authorized agency is no longer obligatory [39]. We can assume that organized tests were a helpful tool to increase the quality of laboratory work and a good source of information for method validation. It is important to highlight that the PT organization, together with workshops and meetings for participants organized every year, allowed to build up the net of MSD laboratories. The presented validation data should be useful for field laboratories and decision-makers. There are limitations of evaluation related to a low number of larvae in samples, and thus we will soon redesign the PT scheme, and our laboratories will be supported with one extra sample spiked with 20 or more larvae.

## 5. Conclusions

This study was aimed to describe parameters characterizing the MSD method in collaborative validation experiments (PT) carried out in a large number of laboratories that allowed to draw the following conclusions:

1. Obtained results confirmed and complemented the validation assumptions presented by Forbes and Gajadhar (1999) [28].
2. MSD methods were characterized by specificity (SP = 97.3%), sensitivity (SE = 86.5%), and accuracy (AC = 89.2%).
3. Limit of detection and limit of quantification were established (LOD = 1 larvae per sample), (LOQ = 3 larvae per sample).

The validation parameters provided in this paper can be useful in the process of implementing new methods, risk assessment and risk management.

The given parameters can be used as a point of reference for other PT organizers in the process of verification of PT programs and in the process of accreditation of individual laboratories.

## Figures and Tables

**Table 1:** Sample qualitative results of PTs from 2015 to 2019 (all levels 0, 1, 3, and 5 larvae).

Year	Number of Laboratories Participating in the Study	Total Number of Sent Samples (Contamination Level 0, 1, 3, 5)	Total Number of Samples Properly Assessed	Total Number of Samples Improperly Assessed	% of Samples Correctly Tested
2015	394	1576	1413	163	89.5
2016	374	1496	1316	180	87.9
2017	384	1536	1390	146	90.5
2018	365	1460	1283	177	87.9
2019	378	1512	1356	156	89.7
Total	1895	7580	6758	822	89.1

**Table 2:** Sample qualitative results of PTs from 2015 to 2019 by the level of contamination.

Contamination Level	Total Number of Samples	Number Evaluated as Incorrect	Number Evaluated as Correct
Level 0	1895	52 (PD)	1843 (NA)
Level 1 *	1895	547 (ND)	1348 (PA)
Level 3	1895	157 (ND)	1738 (PA)
Level 5	1895	66 (ND)	1829 (PA)

Number of false positive (PD), number of true negative results (NA), number of false negative (ND) and number of true positives (PA). * Detection limit—results were not used for evaluation of laboratory performance.

**Table 3:** The statistical description of reported PT results.

Statistical Description	Level 0	Level 1	Level 3	Level 5
Mean	0.05	0.75	2.14	3.51
Standard error	0.01	0.01	0.02	0.03
Median	0.00	1.00	2.00	4.00
Mode	0.00	1.00	3.00	5.00
Standard deviation	0.36	0.54	1.01	1.39
Sample variance	0.13	0.30	1.02	1.94
Kurtosis	137.44	6.66	0.87	−0.22
Skewness	10.56	0.77	−0.39	−0.66
Range	7.00	5.00	9.00	7.00
Minimum	0.00	0.00	0.00	0.00
Maximum	7.00	5.00	9.00	7.00
Sum	92.00	1420.00	4057.00	6646.00
Confidence level (95.0%)	0.02	0.02	0.05	0.06

**Table 4:** Quantitative evaluation of reported results based on |Δ| analysis.

Contamination Level	Total Number of Samples	Number Evaluated as Incorrect	Number Evaluated as Correct
0	1895	52	1843
1	1895	547	1348
3	1895	158	1737
5	1895	426	1469

**Table 5:** Description of validation parameters.

Assessed Parameters	Short Description	Calculation Formula
Precision	Closeness of agreement between independent test results obtained under prescribed conditions.	$\mathrm{CV}=\frac{s}{{x\;}_{\mathrm{mean}}}$ coefficient of variation (CV) is a statistical measure of the relative dispersion of data points in a data series around the mean where s—standard deviation, x_mean_ ≠ 0
Reproducibility	Precision under reproducibility conditions.	$\mathrm{Reproducibility}=\frac{\mathrm{reproduced}\;\mathrm{results}}{\mathrm{total}\;\mathrm{number}\;\mathrm{of}\;\mathrm{samples}}\times 100\%$
Limit of detection	Lowest amount of an analyte to be examined in a test material that can be detected.	LOD = 3 s where s—standard deviation
Limit of quantification	Lowest amount of an analyte to be examined in a test material that can be quantitatively determined.	LOQ = 3 LOD
Sensitivity	EN ISO 16140	$\mathrm{SE}=\frac{\;\left(\mathrm{PA}\right)}{\left(\mathrm{PA}\right)\;+\;\left(\mathrm{ND}\right)}\times 100\%$ The number of true positive results is (PA), and that of false negatives is (ND)
Specificity	EN ISO 16140	$\mathrm{SP}=\frac{\left(\mathrm{NA}\right)}{\left(\mathrm{NA}\right)\;+\;\left(\mathrm{PD}\right)}\times 100\%$ The number of true negatives is (NA), and that of false positives is (PD)
Accuracy	EN ISO 16140	$\mathrm{AC}=\frac{\left(\mathrm{PA}\right)}{\left(\mathrm{PA}\right)\;+\;\left(\mathrm{PD}\right)}\times 100\%$ The number of true positive results is (PA), and that of false positives is (PD)
Uncertainty	ISO/IEC Guide 98–3:2008 Uncertainty of measurement—Part 3: Guide to the expression of uncertainty in measurement (GUM:1995)	(u) = √ [∑ (xi − μ)^2^/(*n* × (*n* − 1))], where the result of the ith PT is (xi), the mean value of PT results is (μ), and the number of results is (*n*)

**Table 6:** Coefficient of variation at different levels.

Reference Value	Level 0	Level 1	Level 3	Level 5
Standard deviations	0.36	0.54	1.01	1.39
X mean	0.05	0.75	2.14	3.51
CV	7.45	0.73	0.47	0.40
ʋ	nm	0.54	0.34	0.28

nm—not marked; ʋ—expected value of measurement

## Data Availability

Not applicable.
